# Underprescription of SGLT2i and GLP-1 RA: CAREPRO-T2D (Cardiorenal Protection in Type 2 Diabetes) Cross-Sectional Study

**DOI:** 10.7759/cureus.33509

**Published:** 2023-01-08

**Authors:** Francisco Simões de Carvalho, Francisca de Brito Marques, Ana Elisa Lopes, Joana Lima Ferreira, Rosa Maria Príncipe

**Affiliations:** 1 Endocrinology, Pedro Hispano Hospital, Matosinhos Local Health Unit, Matosinhos, PRT

**Keywords:** glp-1 receptor agonists, sglt2 inhibitors, atherosclerotic cardiovascular disease, heart failure, type 2 diabetes

## Abstract

Background: New glucose-lowering drugs have shown benefits regarding cardiovascular events, heart failure, and kidney-related outcomes in type 2 diabetes (T2D). This study aimed to estimate the adequacy of SGLT2 inhibitors (SGLT2i) and GLP-1 receptor agonists (GLP-1 RA) prescription to people living with T2D with established atherosclerotic cardiovascular disease (ASCVD) or heart failure (HF).

Material and methods: This was a cross-sectional study based on adults with T2D in a Portuguese local health unit between January 2019 and January 2020. Subjects with ASCVD were compared with subjects without ASCVD, and subjects with HF were compared with subjects without HF regarding clinical and demographic characteristics.

Results: Our study included 13,869 adults with T2D, among whom 5.9% were coded for HF and 20.4% were defined as having ASCVD. SGLT2i were prescribed to 36.0% of subjects with HF. SGLT2i and/or GLP-1 RA were prescribed to 36.1% of patients with ASCVD. When comparing with subjects without ASCVD, subjects with ASCVD were significantly older (70.8 vs. 66.5 years, p<0.001), had lower estimated glomerular filtration rate (68.2 vs. 74.6 mL/min/1.73 m^2^, p<0.001), and higher rates of prescription of SGLT2i and/or GLP-1 RA (36.1 vs. 31.4%, p<0.001). When comparing with subjects without HF, subjects with HF were significantly older (74.6 vs. 66.9 years, p<0.001), had lower estimated glomerular filtration rate (59.6 vs. 74.1, mL/min/1.73 m^2^, p<0.001), and higher rates of prescription of SGLT2i (36.0 vs. 30.3%, p<0.001).

Conclusion: SGLT2i and GLP-1 RA are underprescribed in T2D, with almost two-thirds of patients not being prescribed these agents despite being strongly advised by current guidelines. These findings highlight the need for specific actions to improve T2D management at primary care level.

## Introduction

It is estimated that 13.6% of people aged 20-79 years living with diabetes in Portugal. An average 8.3 years of life are lost per person with diabetes [1]. Besides, diabetes-related financial costs are estimated to account for 0.8% of Portugal’s Gross Domestic Product and 9% of total health expenditure [1]. Type 2 diabetes mellitus (T2D) is by far more prevalent than other types of diabetes [2].

Management of people living with T2D has been changing over the past years due to new data showing that some glucose-lowering drugs have benefits that go beyond glycemic control [3,4]. In light of these events, recommendations regarding pharmacological treatment of T2D have been modified. Recent guidelines recommend that GLP-1 receptor agonists (GLP-1 RA) or SGLT2 inhibitors (SGLT2i) with proven benefits should be used in established atherosclerotic cardiovascular disease (ASCVD) to reduce major adverse cardiovascular events. Also, in people with heart failure (HF), SGLT2i with proven benefits should be used to improve HF and kidney outcomes [5,6]. However, literature shows significant underutilization of these newer organ-protective glucose-lowering agents, especially in primary care settings [7,8]. No data exists regarding prescription of these agents in Portugal.

This study aimed to determine antihyperglycemic therapy prescriptions for people living with T2D in a primary care setting and to study if there were differences in SGLT2i and GLP-1 RA prescription taking into account the presence of ASCVD or HF.

## Materials and methods

Study design, setting, and population

We’ve conducted a retrospective cross-sectional study using data from primary care clinical register platforms in a Portuguese primary care local health unit. Adults who were registered as living with type 2 diabetes between January 2019 and January 2020 were included as per International Classification for Primary Care-2 (ICPC-2) code T90.

Data collection

Subjects’ characteristics were derived from primary care clinical register platform - sex, age, body mass index (BMI), systolic blood pressure (SBP), diastolic blood pressure (DBP), hemoglobin A1c (HbA1c), lipid profile, estimated glomerular filtration rate (eGFR), urinary albumin-to-creatinine ratio, coding for ischemic heart disease (ICPC-2 codes K74, K75, and K76), coding for heart failure (ICPC-2 code K77), coding for atherosclerotic cerebrovascular disease (ICPC-2 codes K89, K90, and K91), and coding for peripheral arterial disease (ICPC-2 code K92). ASCVD was defined as having at least one of the following codes: ischemic heart disease, atherosclerotic cerebrovascular disease, or peripheral arterial disease. Antihyperglycemic medications were obtained from Portuguese electronic prescription platform, an electronic platform owned by the Portuguese Ministry of Health and used nationwide for medical prescription.

Analysis

Subjects’ characteristics are presented as mean (standard deviation {SD}) or n (%). Pearson's chi-square test was used for categorical variables. For continuous variables, Student’s t-test was employed. Results from statistical analyses were accompanied by two-sided 95% confidence intervals and corresponding p-values, with statistical significance defined as p<0.05. SPSS Statistics version 25 (Armonk, NY: IBM Corp.) software was used for statistical analysis.

## Results

Subject characteristics

We have identified 13,869 subjects registered as living with T2D between January 2020 and December 2021, with a mean age of 67.4±11.0 years, a mean BMI of 28.79±4.87 kg/m^2^, and 51.2% were identified as male. Mean HbA1c was 7.1±1.3%. Regarding blood pressure, mean SBP was 136±16 mmHg and mean DBP was 78±10 mmHg. Mean eGFR was 72.2±17.8 mL/min/1.73 m^2^ and median urinary albumin-to-creatinine ratio was 10.4 mg/g.

Coding for ischemic heart disease was present in 1,647 patients (11.9%), coding for HF in 824 patients (5.9%), coding for atherosclerotic cerebrovascular disease in 1,245 patients (9.0%), and coding for peripheral arterial disease in 953 patients (6.9%). In total, 2,834 patients (20.4%) were defined as having ASCVD.

Prescription of antihyperglycemic medication

Metformin was the most prescribed medication (80.7% of patients). Sulfonylureas were prescribed to 17.0% of patients, dipeptidyl peptidase-4 inhibitors (DPP4i) to 38.4%, SGLT2 inhibitors (SGLT2i) to 30.6%, GLP-1 receptor agonists (GLP-1 RA) to 5.6%, pioglitazone and acarbose to 0.7% and 0.6% patients, respectively. Insulin was prescribed to 1,593 patients (11.5%), mainly as basal insulin alone (69.7%). Premixed insulin was prescribed to 18.3% of insulin-treated patients and both basal and rapid-acting insulin to 11.9% of patients.

Subject characteristics according to cardiovascular disease and heart failure

Comparison between patients with or without ASCVD is shown in Table 1. Comparison between patients with or without HF is shown in Table 2.

**Table 1:** Characteristics of the patients according to presence of atherosclerotic cardiovascular disease. HbA1c: hemoglobin A1c, LDL: low-density lipoprotein; GFR: glomerular filtration rate, DPP4i: dipeptidyl peptidase-4 inhibitors; SGLT2i: SGLT2 inhibitors; GLP-1 RA: GLP-1 receptor agonists

Characteristic	ASCVD (N=2834)	No ASCVD (N=11035)	p-Value
Age (years)	70.8±9.6	66.5±11.2	<0.001
Male sex (n, %)	1,818 (64.1)	5,284 (47.9)	<0.001
BMI (kg/m^2^)	28.29±4.70	28.92±4.91	<0.001
HbA1c (%)	7.13±1.31	7.11±1.29	0.601
Systolic blood pressure (mmHg)	136.2±17.2	136.2±15.4	0.843
Diastolic blood pressure (mmHg)	75.5±10.5	78.3±9.9	<0.001
LDL-cholesterol (mg/dL)	83.1±31.4	94.6±30.6	<0.001
Triglycerides (mg/dL)	142.8±87.6	134.0±83.0	0.123
Estimated GFR (mL/min/1.73 m^2^)	68.2±20.1	74.6±16.9	<0.001
Metformin therapy (n, %)	2,183 (77.0)	9,006 (81.6)	<0.001
Sulfonylurea therapy (n, %)	501 (17.7)	1,860 (16.9)	0.299
DPP4i therapy (n, %)	1,244 (43.9)	4,088 (37.0)	<0.001
SGLT2i therapy (n, %)	960 (33.9)	3,288 (29.8)	<0.001
GLP-1 RA therapy (n, %)	190 (6.7)	591 (5.4)	0.005
SGLT2i or GLP-1 RA therapy (n, %)	1,022 (36.1)	3,464 (31.4)	<0.001
Insulin therapy (n, %)	521 (18.4)	1,072 (9.8)	<0.001

**Table 2:** Characteristics of the patients according to presence of heart failure. HbA1c: hemoglobin A1c, LDL: low-density lipoprotein; GFR: glomerular filtration rate, DPP4i: dipeptidyl peptidase-4 inhibitors; SGLT2i: SGLT2 inhibitors; GLP-1 RA: GLP-1 receptor agonists

Characteristic	HF (N=824)	No HF (N=13,045)	p-Value
Age (years)	74.6±9.4	66.9±10.9	<0.001
Male sex (n, %)	411 (49.9)	6691 (51.3)	0.431
BMI (kg/m^2^)	29.45±5.40	28.75±4.83	<0.001
HbA1c (%)	7.14±1.37	7.12±1.29	0.665
Systolic blood pressure (mmHg)	133.1±17.9	136.4±15.6	<0.001
Diastolic blood pressure (mmHg)	73.2±11.1	78.0±10.0	<0.001
LDL-cholesterol (mg/dL)	83.2±30.4	92.8±31.1	<0.001
Estimated GFR (mL/min/1.73 m^2^)	59.6±21.0	74.1±17.2	<0.001
Metformin therapy (n, %)	560 (68.0)	10,629 (81.5)	<0.001
Sulfonylurea therapy (n, %)	117 (14.2)	2,244 (17.2)	0.026
DPP4i therapy (n, %)	375 (45.5)	4,957 (38.0)	<0.001
SGLT2i therapy (n, %)	297 (36.0)	3,951 (30.3)	0.001
GLP-1 RA therapy (n, %)	67 (8.1)	714 (5.5)	0.001
Insulin therapy (n, %)	164 (19.9)	1,429 (11.0)	<0.001

## Discussion

Notwithstanding the growing body of evidence displaying cardiorenal benefits for SGLT2i and GLP-1 RA therapies [3,4], studies have shown their prescription to be suboptimal, as did ours [7,8]. Only 36.1% of patients with ASCVD are under SGLT2i or GLP-1 RA or both therapies combined, and just 36.0% of patients with HF are under SGLT2i.

These new agents are considerably more expensive than other older drugs, like metformin or sulfonylureas. However, the Portuguese National Health Service strongly facilitates people’s access, covering 90-95% of economic costs of all non-insulin glucose-lowering drugs to people living with diabetes. Several other reasons can explain SGLT2i and GLP-1 RA subprescription.

Therapeutic inertia has been recognized as a major problem in T2D management and changing drug treatment demands a proactive approach by both patients and physicians [9]. Mean HbA1c in all groups stands at around 7.1%, slightly above the recommended target of 7.0%. One could argue that many people will be adequately treated glycemic-wise. However, the benefits of SGLT2i and GLP-1 RA are largely independent of their glucose-lowering effects [3,4]. GLP-1 RA therapy prescription in ASCVD is extremely low, at under 7%. One of the reasons could be that in Portugal only injectable GLP-1 RA are available, and this route of administration might limit their use by either patient-centered or physician-centered barriers to injectable therapy. Nevertheless, this fact wouldn’t explain such low prescriptions as over 15% of these patients are under insulin therapy, also administered as subcutaneous injection. More emphasis must be put on updating primary care clinicians’ knowledge regarding T2D and on building multidisciplinary teams involving endocrinologists and primary care health professionals for community-based T2D management programs [10].

DPP4i are widely used despite their moderate efficacy and not having proven cardiorenal benefits. Also, no generic preparations of DPP4i existed at the time of the study, making their economic cost similar to SGLT2i’s. Surprisingly, they’re prescribed to over a third of patients and their prescription is higher in patients with ASCVD and HF, even above SGLT2i or GLP-1 RA. Clinical inertia could justify using older DPP4i instead of SGLT2i or GLP-1 RA. Still, SGLT2i in HF and SGLT2i and GLP-1 RA in ASCVD are significantly more prescribed than in the absence of these complications. Hopefully, this could lead to a change in prescription trends and an improvement might be observed over the next years.

Metformin is the most prescribed drug, as expected, in line with guidelines dated from the time of the study [11]. However, probably related to lower eGFR, its prescription is significantly lower in patients with ASCVD and HF. Insulin therapy is prescribed more frequently to patients with ASCVD and HF when compared to patients without these complications. Lower eGFR might help to explain this fact, as would older age and the possible coexistence of other complications.

One important fact regarding this population is the high risk for cardiovascular events. As such, mean low-density lipoprotein-cholesterol (LDL-cholesterol) levels are unacceptable by current standards [6,12,13]. Certainly, there seems to be an effort directed at LDL-cholesterol lowering in ASCVD, as mean levels are lower. However, mean LDL-cholesterol levels at 83.1 mg/dL are substantially higher than recommended <55 mg/dL for these patients. We highlight the need for holistic management of T2D with strategies to optimize cardiovascular risk factors. Also, coverage of statin and ezetimibe therapy by the Portuguese National Health Service (NHS) at the same level as antihyperglycemic drugs could lead to better outcomes.

Our study has some relevant limitations. There is substantial lack of data regarding some important points, such as patient’s socioeconomic status or time from T2D diagnosis. The retrospective nature of the study makes it based solely on clinical records and coding, and important data could be missed. A future prospective study could eliminate some of these biases and produce dynamic results.

## Conclusions

Our study shows substantial underprescription of SGLT2i and GLP-1 RA in T2D with ASCVD or HF. Almost two-thirds of patients are not being prescribed cardiorenal-protective agents despite being strongly advised by current guidelines and recommendations.

Special programs must be employed involving primary care health professionals and endocrinology specialists in order to provide the best care available to people living with T2D, particularly when there is target-organ damage. Economic cost of drugs for T2D management is not a significant barrier in our setting, given the substantial Portuguese National Health Service coverage, indicating that other limitations are as or more important.
